# Evaluation of Cardiac Arrhythmias before, during, and after Treadmill Exercise Testing in Poorly Performing Standardbred Racehorses

**DOI:** 10.3390/ani11082413

**Published:** 2021-08-16

**Authors:** Elena Alberti, Luca Stucchi, Chiara Maria Lo Feudo, Giovanni Stancari, Bianca Conturba, Francesco Ferrucci, Enrica Zucca

**Affiliations:** 1 Equine Sports Medicine Laboratory “Franco Tradati”, Department of Veterinary Medicine, Università degli Studi di Milano, 26900 Lodi, Italy; elena.alberti@unimi.it (E.A.); chiara.lofeudo@unimi.it (C.M.L.F.); francesco.ferrucci@unimi.it (F.F.); 2 Veterinary Teaching Hospital, Università degli Studi di Milano, 26900 Lodi, Italy; luca.stucchi@unimi.it (L.S.); giovanni.stancari@unimi.it (G.S.); bianca.conturba@unimi.it (B.C.)

**Keywords:** premature complexes, Holter recording, Standardbred trotters, poor performance, plasma lactate

## Abstract

**Simple Summary:**

In sport horses, cardiovascular problems, such as arrhythmias, can cause poor performance. However, only the role of atrial fibrillation and severe bradyarrhythmias on athletic performance has been well established. On the other hand, the significance of other arrhythmias, such as supraventricular and ventricular premature complexes, is still not well defined. The present study reports on the prevalence of cardiac arrhythmias during maximal treadmill exercise in poorly performing Standardbreds, and investigates the possible relationship of demographic, cardiac, and performance indices on premature complexes. The results showed weak evidence of the dependence of premature complexes on minimum heart rate, and a tendency for the occurrence of premature complexes to increase with aging and increasing maximum lactate concentration. Our results suggest that premature complexes are frequent in poorly performing Standardbred racehorses, but further studies are necessary to clarify their role and clinical significance.

**Abstract:**

The incidence of significant arrhythmias in sport horses and knowledge about their exact influence on athletic performance need to be clarified. The aims of the present study are to report the prevalence of cardiac arrhythmias during maximal treadmill exercise in poorly performing Standardbreds, and to investigate the possible relationship of demographic, cardiac and performance indices on premature complexes (PCs). Electrocardiographic Holter recordings before, during and after treadmill exercise testing of 158 poorly performing Standardbreds were analyzed retrospectively. Fifty horses did not have any type of arrhythmia. One hundred and eight horses had at least one type of arrhythmia, such as sinus arrhythmia (8.2%), sinoatrial block (3.2%), second-degree atrioventricular block (33.5%), supraventricular PCs (7.6%), and ventricular PCs (48.1%). A multiple regression analysis showed weak evidence that the occurrence of premature complexes decreases as the minimum heart rate increases, and a tendency for these arrhythmias to increase with increasing age and maximum lactate concentration. Our results suggest that PCs are frequent in poorly performing Standardbred racehorses, but further studies are necessary to clarify their role and clinical significance.

## 1. Introduction

In sport horses, cardiovascular problems represent the third cause of poor performance after musculoskeletal and respiratory disorders [1]. Among these, arrhythmias are of particular interest. Atrial fibrillation is a common arrhythmia in performance horses and its influence on athletic performance has been established [2]. Moreover, it is reported that horses with severe bradyarrhythmias (advanced second-degree and third-degree atrioventricular blocks) may have severe exercise intolerance and collapse [3]. On the other hand, the significance of other arrhythmias, such as supraventricular and ventricular premature complexes (PCs), is still not well defined. Martin et al. (2000) suggested that premature complexes have to be considered clinically important if more than two isolated PCs were detected during maximal exercise or if multiple PCs (>5) or pairs or paroxysms of PCs were detected during peak exercise or immediately after exercise [4]. During the last fifteen years, different studies have investigated the possible influence of PCs on equine athletic performance. Particular interest was focused on racehorses [5,6,7,8,9,10,11,12,13] in which even slight variations of cardiac rhythm may play an important role [1]. Recently, different authors have reported data about the prevalence of arrhythmias in normal performing showjumping horses [14,15], dressage horses [16], reining horses [17], and competition draft horses [18].

According to the current knowledge, the guidelines suggested by Martin et al. [4] are under discussion, and different authors agree that these arrhythmias could be observed in a significant percentage of clinically normal, well performing sport horses [5,6,14,16].

Nowadays, it might be better to refer to the ACVIM/ECEIM (American College of Veterinary Internal Medicine and European College of Equine Internal Medicine) consensus statement for the management of athletic horses with cardiovascular abnormalities [19]. Some recommendations for equine athletes are quite similar to those reported for human competitive athletes with PCs [20]. In particular, supraventricular premature complexes (SVPCs) in the absence of structural heart disease and symptoms are not considered dangerous for the athletes, both human and equine. Instead, when ventricular premature complexes (VPCs) or other ventricular arrhythmias occur, a more in-depth evaluation is suggested [19,20]. Therefore, the exact influence of PCs on athletic performance, particularly when they are infrequent, isolated and/or detected in periods different from peak exercise, should be further investigated.

The aims of the present study are to report the prevalence of cardiac arrhythmias detected before, during and after maximal treadmill exercise in poorly performing Standardbred racehorses, and to investigate the possible impact of some demographic, cardiac, and performance indices on PCs in poor performing racehorses. The indices considered are sex and age, cardiac indices such as minimum heart rate (HRmin), maximum heart rate (HRmax), the difference between the maximum heart rate and the heart rate at 1 min of recovery (delta-HR), and performance indices such as maximum speed (Vmax), maximum plasma lactate concentration (lactate_max_), and time to fatigue.

## 2. Materials and Methods

### 2.1. Horses

In this study, data were collected retrospectively from the medical records of Standardbred trotter racehorses with poor performance admitted to the Equine Internal Medicine and Sports Medicine Unit of the University of Milan, between January 2005 and December 2015, for clinical and instrumental evaluations at rest and during exercise on high-speed treadmill. An informed client consent was signed by owners as part of the routine hospital admission policy. Clinical data were used in accordance with international legislation regarding ethical animal research, which does not require a submission of the research protocol to the Institutional Animal Care and Use Committee for retrospective study. The study population was selected according to the following inclusion criteria: the results of a (1) thorough physical examination to rule out obvious causes of poor performance at rest, (2) plasma lactate curve obtained during a treadmill incremental exercise test, and (3) continuous Holter monitoring before, during, and after high-speed treadmill test, were available.

### 2.2. Treadmill Exercise Testing and Holter Recording

#### 2.2.1. Treadmill Test Protocol 

During the treadmill test, horses were dressed in a racing harness and a safety belt. Horses acclimatized on a high-speed treadmill (SATO-1, Sweden) underwent an incremental test to obtain a plasma lactate curve. The treadmill test was performed with a slope of 3°. The standardized protocol consisted of a warm-up period of 4 min at a walk (1.5 m/s) and 3 min at a trot (6 m/s), and then the treadmill speed was increased by 1 m/s every minute from 7 m/s until the horse became unable to maintain the belt speed [21,22]. At this time, the belt speed was rapidly decreased to 1.5 m/s. During the recovery phase, horses were maintained at a walk on the treadmill with a slope of 0° until the heart rate decreased to 100 bpm.

#### 2.2.2. Plasma Lactate Curve

Samples were collected from the jugular vein by means of a 14G Teflon venous catheter connected to an extension tube. After that, samples were immediately transferred into tubes with potassium fluoride. In order to obtain the lactate curve, samples were taken at rest, at the end of the warm-up period at a trot, at the end of each incremental step, and during the recovery period at 1, 5, 15, and 30 min after the end of the exercise [21]. Lactate concentration was determined on plasma samples. The samples were analyzed by the enzymatic-colorimetric technique (Lactate Dry-Fast, SENTINEL Diagnostics, Milan, Italy) using a photometer (INSTRUMENTS SCLAVO^®^ Unifast system 2 analyzer, Siena, Italy). After 10 min of incubation at 37 °C, the samples were read at 540 nm.

#### 2.2.3. Heart Rate

During the treadmill exercise testing the heart rate was monitored using a heart rate monitor (POLAR^®^ EQUINE inzone FT1, Steinhausen, Switzerland). Electrodes were positioned on the left chest wall caudally to the withers (positive) and behind the olecranon (negative).

#### 2.2.4. The Treadmill Test’s Parameters 

The following parameters were taken from data obtained during the treadmill test:

- V_200_: speed at which the heart rate reached 200 bpm;
- Vmax: maximum speed reached by the treadmill;
- time to fatigue: from the beginning of the first incremental step (7 m/s) to the end of the test;
- lactate_max_: maximum plasma lactate concentration;
- HRmin: minimum heart rate at rest;
- HRmax: maximum heart rate reached during the test;
- HR1min: heart rate at 1 min post exercise during the recovery phase. The time point used to mark the end of exercise was the moment the speed was reduced on the treadmill. The time recorded by a chronometer during the treadmill test and the time superimposed on the ECG recording were matched, and the end of exercise was marked on the ECG;
- delta-HR: Difference between the maximum heart rate and the heart rate recorded at 1 min post exercise.

#### 2.2.5. ECG Holter Equipment and the Recording Method

**Figure 1:**
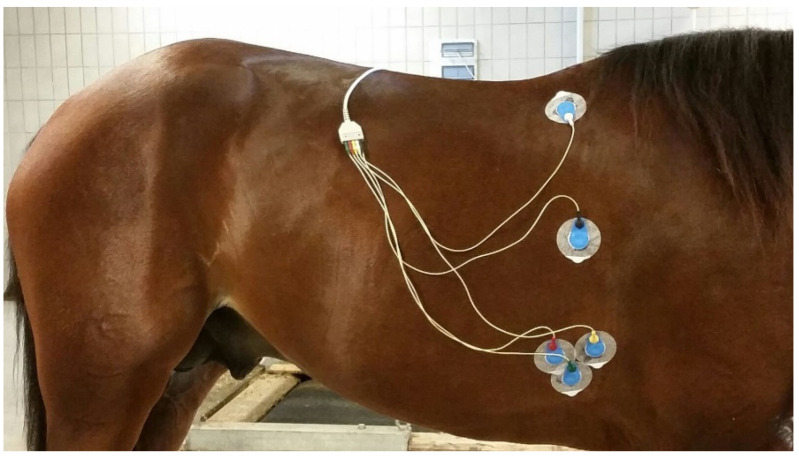
Positioning of the electrodes according to a modified base-apex configuration; reference electrode white; earth electrode black; exploring electrodes red, yellow, and green.

The ECG was obtained using a Holter recorder (CARDIOLINE^®^ CLICK Holter, Trento, Italy), which recorded 3 unipolar leads. A modified base-apex configuration was used as previously described [22]. Electrodes were positioned on the right chest wall caudally to the withers (reference electrode), behind the olecranon (3 exploring electrodes), and the earth electrode between those previously described (Figure 1). This placement of electrodes gave three identical leads. Electrodes and connecting cables were held in position by a chest belt (Vetrap^®^ 3M, Roma, Italy). The recorder was then fixed on the safety harness. The ECG signal was recorded continuously before exercise (15 min), during the incremental test, and during the post-exercise recovery period (30 min).

### 2.3. ECG Analysis

Data were analyzed using dedicated software (CLICK Holter CARDIOLINE^®^—PRIMA manager, Trento, Italy). Since the software is set for the use in human medicine, and therefore could be inaccurate in equine medicine, all ECG recordings were analyzed manually. The quality of each ECG recording was assessed by an inspection of the whole recording. Where all the ECG leads were unreadable/missed for a variable time, the recording quality was considered unsatisfactory, and the horse was excluded from the study. An experienced operator, annotating the type and the period of occurrence (rest, warm-up, sub-maximal exercise, peak exercise, 1 min of recovery, and late recovery) recorded the presence of any arrhythmia [22]. Rest was considered the period of recording prior to the treadmill test. The warm-up period was defined as the treadmill phase at a walk and at a trot at 6 m/s. The sub-maximal exercise period included the phases of the incremental test until the V_200_ was reached. Peak exercise referred to the phases of the incremental test from V_200_ to the end of maximal exercise. The 1 min of the recovery phase was considered the first minute after the end of the treadmill test. Late recovery was described as the following phase of the ECG recording. 

Sinus arrhythmia was defined as variable RR intervals, and where waxing and waning of the heart rhythm was evident; the QRS complex configuration and the relation between the P wave and the QRS complex were normal [23,24]. Sinoatrial (SA) block was recognized as the absence of the P wave and the QRS complex for a duration of twice the previous PP interval [23,24]. Second-degree atrioventricular (second-degree AV) block was diagnosed if the P wave was not associated with any following QRS complex with a double length of the RR interval [3,23,25]. The supraventricular premature complex was identified as an individual premature complex characterized by the presence of a QRS complex of normal configuration and followed by a non-compensatory pause [23,25]. Supraventricular premature complexes post-exercise were distinguished from sinus arrhythmia when an underlying stable heart rate was interrupted by intermittent premature complexes [5]. The ventricular premature complex was defined as an individual premature QRS and T complex with a configuration that differed from that of the sinus QRS and T complexes [23,24,25]. A QRS complex, not preceded by a P wave, was considered abnormal when changes in the relative size of the Q, R, or S waves produced changes in the configuration of the complex, or when the duration of the QRS complex varied [26]. Most commonly, ventricular premature complexes were followed by a compensatory pause [23,24,25]. Supraventricular and ventricular complexes were considered premature when the RR interval decreased by more than 20% (during rest) or 10% (during exercise) at a distance from the previous RR interval [8,14,16]. Supraventricular and ventricular tachycardia were characterized as abnormal rhythms caused by 3 or more repetitive or linked SVPCs or VPCs respectively [23].

### 2.4. Statistical Methods

#### 2.4.1. Descriptive Analysis

The prevalence of each type of arrhythmia (sinus arrhythmia, SA block, second-degree AV block, SVPCs, VPCs) in the entire recorded ECG and in each period of recording was calculated as the number and the percentage of horses. 

Concerning PCs, the number of horses with isolated, pairs, and episodes of supraventricular and ventricular tachycardia were reported. Moreover, the number of SVPC and VPC episodes per horse were calculated as the range, median, mean, and standard deviation.

Data distribution was tested for normality using the Shaphiro–Wilk test. Normally distributed data are presented as mean and standard deviation. Non-normally distributed data are presented as median and interquartile range. Categorical data are presented as numbers and percentages.

#### 2.4.2. Inference Statistical Analysis

Inference statistical analysis was conducted in three steps. Firstly, a univariate logistic regression analysis was used to identify statistically significant (*p* < 0.25) independent variables (sex, age, HRmin, HRmax, delta-HR, Vmax, lactate_max_, and time to fatigue) associated with the two possible outcomes. The outcomes considered were “at least one” and “severe arrhythmias”. “At least one” was classified as the presence of at least one PC (SVPC and/or VPC) in any period of recording. “Severe arrhythmias” were classified as the presence of >2 isolated PCs detected during peak exercise or multiple PCs (>5) or pairs or paroxysms of PCs detected during peak exercise or immediately after exercise [4]. Then, a multiple logistic regression analysis was conducted to identify the independent variables that were significantly (*p* < 0.05) associated with the outcomes to be included in the inference multivariable model. Eventually, a multiple logistic regression analysis was conducted. All analyses were performed by means of R software.

## 3. Results

### 3.1. Horses

Among 414 poorly performing Standardbred trotter racehorses admitted, 161 horses met the inclusion criteria. Three horses were subsequently excluded because of unsatisfactory ECG recording quality. Accordingly, 158 horses were included in the study.

Among these, 80 (50.6%) were males, 10 (6.3%) were geldings, and 68 (43.1%) were mares. The median age was 3 years old (IQR 1 year; range 2–8 years old).

### 3.2. Prevalence of Arrhythmias 

Fifty horses (31.6%) did not have any type of arrhythmia, while 108 subjects (68.4%) had at least one type of arrhythmia in one or more phases of the exercise testing. In particularly, 5 horses (3.2%) had SA block; 13 horses (8.2%) had sinus arrhythmia; 12 horses (7.6%) had SVPCs; 53 horses (33.5%) had second-degree AV block; and 76 horses (48.1%) had VPCs (Table 1).

Sinus arrhythmia was recorded at rest, during the first minute of recovery or during the late phase of recovery post exercise, while it was never detected during the sub-maximal and peak exercise periods. SA blocks were observed particularly at rest, while no horses experienced SA block during the warm-up, sub-maximal and peak exercise periods. Most of the second-degree AV blocks were observed at rest and during the first minute of recovery. No horses had this type of arrhythmia during the sub-maximal and peak exercise phases.

Eighty-two horses (51.9%) had PCs (supraventricular and/or ventricular). In particular, 6 horses (3.8%) had SVPCs but no VPCs, 70 horses (44.3%) had VPCs but no SVPCs, while 6 horses (3.8%) had both SVPCs and VPCs. Most of the SVPCs (4 horses) and VPCs (66 horses) were observed during the first minute post-exercise; in a lower number of subjects, PCs were detected during peak exercise (SVPCs no case; VPCs four cases). Premature complexes were mainly observed as single or isolated premature beats; in particular, the number of SVPCs recorded per horse ranged from 1 to 36 events, while the number of VPCs observed per horse ranged from 1 to 39 events. Ventricular premature complexes were also detected as pairs in 21 horses with the number of coupled VPCs per horse ranging from 1 to 10. 

In four horses, transient episodes of ventricular tachycardia were recorded post-exercise. In these cases, only one episode per horse was observed (Table 2).

### 3.3. Treadmill Test Parameters

During the treadmill incremental test, the horses reached a maximum speed median value of 11 m/s (IQR 1 m/s). The minimum speed value was 9 m/s and the maximum was 12 m/s. The median time to fatigue value, calculated from the beginning of the first incremental step to the end of the test, was 3 min and 10 s with an IQR of 1 min and 12 s (ranging from 1 min and 29 s to 6 min). The median V_200_ value was 7 (IQR 2 m/s), with a maximum V_200_ of 10 m/s recorded in seven horses. Lactate_max_ ranged between 9.32 mmol/L and 41.85 mmol/L (mean 24.85 ± 6.60 mmol/L). The peak of lactate concentration was reached during the last incremental step or at 1, 5, or 15 min of recovery, with a prevalence of 67.1% at 5 min post-exercise. The HRmin ranged between 22 bpm and 64 bpm (median 32 bpm; IQR 5 bpm). The HRmax ranged between 203 bpm and 241 bpm (median 231 bpm; IQR 11 bpm). The mean HR1min was 154 ± 19 bpm (range 100–193 bpm). A great variability in the delta-HR value was observed; in fact, this value, calculated as the difference between the HRmax and the HR1min, ranged between 138 bpm and 32 bpm (mean 76 ± 18 bpm). 

### 3.4. Statistical Analysis

According to the type of arrhythmia, 82 horses were grouped in the “at least one” classification, while 76 horses had no PCs. Demographic, cardiac, and performance data are presented in Table 3.

According to the type, number, and timing of the arrhythmias, 23 horses were grouped in the “severe arrhythmias” classification, while 135 horses had no severe arrhythmias. In particular, in three horses the presence of more than two PCs was observed during the maximal exercise period. One horse had multiple PCs (>5 PCs) during the first minute of recovery. Seventeen subjects had coupled PCs during the first minute of recovery, while in two horses paroxysms of ventricular tachycardia were recorded in the first minute of the recovery treadmill phase. Demographic, cardiac, and performance data are reported in Table 4.

Regarding the “at least one” outcome classification, the analysis of the final multiple logistic regression model (Table 5) showed no significant relationship between the independent variables and the occurrence of the PCs. A tendency of PCs to increase with increasing age was observed.

In the case of the “severe arrhythmias” outcome classification, the analysis of the final multiple logistic regression model showed weak evidence of the dependence of the occurrence of PCs on HRmin. The OR estimate was lower than 1, so the event odds tended to decrease as HRmin increased. Moreover, a tendency of PCs to increase with increasing lactate_max_ was found (Table 5).

## 4. Discussion

Arrhythmias detected before, during, and after an incremental treadmill test in poorly performing Standardbreds in the present study seem to be quite common. Interestingly, in addition to bradyarrhythmias, such as SA block (3.2%), second-degree AV block (33.5%), and sinus arrhythmia (8.2%), the data showed a high prevalence of PCs (51.9%) in these horses. In particular, the analysis of the prevalence of arrhythmias in the different periods of recording revealed that bradyarrhythmias were mainly observed at rest and during the first minute of recovery, while the PCs were recorded in a few cases at rest and during the warm-up period, but also at maximal exercise and during the first minute of recovery. 

As reported in the literature, the prevalence of sinus arrhythmia (64.8%) is higher in Thoroughbreds, observed during training sessions, while second-degree AV blocks are less frequent (16.2%) [5]. The difference in the data obtained in the present study may be due to the recording conditions (on a treadmill exercise in a quiet environment vs. during a training session on a racetrack). In fact, during training the presence of other subjects, racetrack conditions, and other factors may induce wider autonomic tone fluctuations and the maintenance of a higher heart rate at rest that may have led to a higher prevalence of sinus arrhythmia and to a lower prevalence of second-degree AV blocks. 

The total prevalence of PCs in our study seems to be quite similar to those reported in a study conducted on poorly performing Thoroughbred racehorses, even if the results obtained considering SVPCs and VPCs separately are quite different. The prevalence of PCs (supraventricular and/or ventricular) recorded in Thoroughbreds during the treadmill exercise was 63%; VPCs were observed in 26% of horses, 19% of the subjects had only SVPCs, and 17% had at least one SVPC and one VPC [6]. Moreover, the main incidence of the PCs was recorded during the high-speed phases and immediately after the maximal exercise, as in the present study. 

A significant prevalence of PCs has also been reported in normal [7,8,9] and poorly performing Standardbred racehorses [11] and in poorly performing Thoroughbreds [13]. A comparison of the data is not always possible due to the different conditions and timing of the recording of the arrhythmias. A recent study reported a high prevalence of PCs in poorly performing Standardbred during the warm-up (7.8%) and high-speed exercise periods (43%) of a high-speed exercise test [11]. In a large observational study, the presence of ventricular arrhythmias was recorded immediately post-exercise in 27.8% of horses, but no arrhythmias were observed during the race [7]. Moreover, a high prevalence of SVPCs during (50%) and immediately after racing (46.2%), and a more modest incidence of VPCs (during 3.8% and immediately after racing 19.2%) were tested for a possible association with heart size, valvular regurgitations, and cardiac Troponin I concentration in normal performing Standardbreds [9]. According to the absence of a significant association between arrhythmias and any of the parameters considered, the authors concluded that the presence of isolated SVPCs and VPCs, even in large numbers, had to be considered physiological in origin [9].

Given the high prevalence of these arrhythmias in both normal and poorly performing racehorses, the observation of isolated PCs alone cannot explain why a horse shows a decline in athletic performance. Accordingly, the role of premature complexes remains to be defined.

Our results only showed a tendency of the occurrence of PCs to increase with increasing age; a similar but a statistically significant result was previously observed in normal Standardbreds [9]. From a general point of view, it is well known that long-term training induces a heavy cardiovascular remodeling, and that the cardiac eccentric hypertrophy makes equine athletes more susceptible to atrioventricular valve insufficiencies [9,27,28]. It might be suggested that even an increase in the prevalence of PCs with aging could be related to the cumulative effect of long-term training and cardiac changes in the athlete [9].

As the “severe arrhythmias” outcome classification was considered, weak evidence that the occurrence of PCs decreases as HRmin increases, and a tendency for PCs to increase with an increase in lactate_max_ was observed in our study.

A similar relationship was described in poorly performing Thoroughbred racehorses [6] and in poorly performing horses of different breeds [10] exercising on a treadmill. In particular, the study conducted on poorly performing Thoroughbreds reported a significant higher blood lactate concentration, at 2 min post-exercise, in horses with PCs [6]. The second study reported an association between peak lactate and arrhythmias occurring during peak exercise [10]. Our results show only a tendency to a positive effect of increasing lactate_max_ on PCs occurrence, and therefore, no substantial clinical interpretations may be made. However, from a general point of view hypoxia and electrolyte disturbances, which could occur during strenuous exercise, may cause ventricular arrhythmias both during and after maximal exercise [5]. On the other hand, premature beats, particularly during exercise, may produce a reduced cardiac output and consequently a reduced muscle perfusion, inducing a premature hypoxia. In both cases, an earlier transition to the anaerobic metabolism may induce an increase in lactate production.

We did not evaluate the possible association between the causes of poor performance, particularly upper or lower airways diseases, and PCs in the studied population, and this should be considered as a potential limitation of our study. However, no significant relationships were found between premature complexes and diagnosis of airway dysfunction (dorsal displacement of the soft palate, palatal instability, or other respiratory problems of upper and lower airways) in poorly performing Thoroughbred racehorses [6], in poorly performing Standardbred and Norwegian–Swedish Coldblooded trotters [11] and in poorly performing horses of different breeds [10]. Conversely, a recent study found an association between an ectopic/re-entrant rhythm and an upper airway obstruction in poorly performing Thoroughbreds [13]. The exact role of dynamic upper airway obstruction and arterial hypoxemia on the development of PCs has not been clearly established [6]. Further studies would be necessary to investigate the possible association between the occurrence of significant arrhythmias and the specific cause of reduced athletic ability in poorly performing horses.

Another limitation of this study could be the use of a single lead that could make more difficult to differentiate between normal and abnormal complexes [29]. This could have biased the prevalence counts of the SVPCs and VPCs but cannot have introduced an error in the statistical analysis because the SVPCs and VPCs were considered together as PCs. According to the high prevalence of the isolated PCs both in normal and in poorly performing racehorses, and the observation that the role of premature complexes remains to be defined, a deep revision of the criteria indicating significant arrhythmias is mandatory [5,6,14,16].

Nowadays, we can refer to the ACVIM/ECEIM consensus statement for the management of athletic horses with cardiovascular abnormalities [19]. ACVIM/ECEIM recommendations state that SVPCs are an uncommon cause of poor performance, and horses with occasional SVDPs, even if during exercise, are considered as safe to ride or drive as their age-matched peers. Nevertheless, frequent SVPCs should be considered as a risk factor for the onset of atrial fibrillation [19].

More concern is expressed about the definition of risks to the horse with ventricular arrhythmias. The analysis of VPCs’ morphology, number (isolated, coupled, or paroxysm), frequency, distributional pattern, and prematurity, might be useful to evaluate their clinical significance [19].

The ACVIM/ECEIM consensus statement considers that occasional monomorphic VPCs occurring during or immediately post exercise, frequently reported in normal performing horses, are not usually a cause for poor performance [19].

The presence of premature complexes after maximal exercise is generally considered an incidental finding [5] or of no clinical significance, as it is not related to structural cardiac alterations nor to an increase in myocardial damage indicators [8]. Nevertheless, different authors stated that their role as a risk factor for the development of sudden death, immediately after intense exercise in athletic horses, requires further investigation [7,8,19]. Moreover, their relationship to poor performance is uncertain and further studies are necessary [19].

Additional investigations (echocardiogram and 24 h Holter monitoring) are recommended for horses with ventricular tachycardia or complex ventricular arrhythmias. When VPCs are recurrent or identified in the clinical settings of poor performance, an exercising ECG should be included in the evaluation protocol [19].

Concerning human sport medicine, eligibility recommendations for competitive athletes with cardiovascular abnormalities have attempted to establish prudent guidelines to manage cardiovascular diseases. In fact, these diseases place the competitive athlete at increased risk for sudden and unexpected death or disease progression [20]. Regarding PCs, such recommendations are quite similar to those reported for equine athletes. In particular, athletes with premature atrial complexes in the absence of structural heart disease and symptoms can participate in all competitive sports. When ventricular premature complexes or other ventricular arrhythmias occur, more of an in-depth evaluation is suggested and recommendations may differ according to the type of arrhythmia and sport. For example, athletes with structural heart disease or athletes with symptoms or an increase in VPCs during exercise can participate only in very low intensity competitive sport [20].

## 5. Conclusions

In conclusion, the data reported in the present study suggest that PCs are frequently recorded in poorly performing Standardbred racehorses.

Given the small number of horses with “severe arrhythmias” detected in the present retrospective study, further prospective research on a larger number of horses is needed to better clarify our results, and to confirm or refute the tendency for PCs to increase with the increasing in lactate_max_ and aging.

The results obtained in the present study did not clarify the role and clinical significance of premature complexes in poorly performing horses. In fact, we cannot rule out a possible influence of concomitant problems (causes of poor performance) on our results. Nevertheless, we would like to suggest that the presence of PCs in racehorses, recorded during and after strenuous exercise, should be further investigated.

## Figures and Tables

**Table 1:** Distribution of rhythm disturbances during each period of recording in 158 Standardbred trotter racehorses.

Event	All ECG Recordings		Rest		Warm-Up		Sub-Maximal Exercise		Maximal Exercise		1 Minute of Recovery		Late Recovery	
	n.	%	n.	%	n.	%	n.	%	n.	%	n.	%	n.	%
NSR	50	31.6	116	73.4	151	95.6	158	100	154	97.5	94	59.5	134	84.8
Sinus arrhythmia	13	8.2	3	1.9	1	0.6	0	0	0	0	8	5.1	2	1.3
SA block	5	3.2	4	2.5	0	0	0	0	0	0	1	0.6	1	0.6
Second-degree AV block	53	33.5	29	18.4	1	0.6	0	0	0	0	29	18.4	8	5.1
SVPC	12	7.6	6	3.8	2	1.3	0	0	0	0	4	2.5	1	0.6
VPC	76	48.1	7	4.4	4	2.5	0	0	4	2.5	66	41.8	13	8.2

NSR: normal sinus rhythm; SA block: sinoatrial block; Second–degree AV block: second-degree atrioventricular block; SVPC; supraventricular premature complex; VPC: ventricular premature complex; n.: number of horses in which the event was observed; %: percentage of horses in which the event was recorded.

**Table 2:** Distribution of rhythm disturbances, recorded in 158 Standardbred trotter racehorses, expressed as the number of complexes per horse.

	All Events			Isolated				Pairs				Paroxysm of PCs			
	Range	Median	Mean ± SD	n.	Range	Median	Mean ± SD	n.	Range	Median	Mean ± SD	n.	Range	Median	Mean ± SD
SVPC	1–39	1.5	6 ± 10.8	12	1–36	1.5	5.7 ± 10	1	3	3	3 ± 0	0	0	0	0
VPC	1–41	2	4.2 ± 6.2	69	1 -39	2	4.2 ± 6.1	21	1–10	1	2.1 ± 2.1	4	1	1	1 ± 0

PCs: premature complexes; SVPC: supraventricular premature complex; VPC: ventricular premature complex; n.: number of horses in which the event was recorded; range: minimum value-maximum value; median: median value; mean: mean value; SD: standard deviation.

**Table 3:** Demographic, cardiac, and performance data of horses grouped according to the “at least one” classification.

**Variable**		**“At Least One” Horses**		**No PCs Horses**	
		**n**	**%**	**n**	**%**
Sex	female	32	47	36	53
	gelding	5	50	5	50
	male	45	56	35	44
**Variable**		**mean ± SD**	**median (IQR)**	**mean ± SD**	**median (IQR)**
Age (y.o)		-	4 (2)	-	3 (1)
HRmin (bpm)		-	32 (7)	-	31 (5)
HRmax (bpm)		-	232 (11)	-	231 (11)
HR1min (bpm)		154 ± 19	154 (19)	153 ± 19	153 (25)
Lactate_max_ (mmol/L)		-	26.56 (6.68)	24.07 ± 6.90	24.17 (9)
Vmax (m/s)		-	11 (1)	-	11 (1)
Time to fatigue (s)		-	195 (74)	-	182 (49)
Delta-HR (bpm)		-	74 (18)	76 ± 18	76 (24)

The table reports the number and percentage of horses with at least one PC and horses with no PCs (categorical variables), the mean and standard deviation values (continuous variables normally distributed) and the median and interquartile range (continuous variables normally and non-normally distributed). PC: premature complex; n.: number of horses; %: percentage of horses; SD: standard deviation; IQR: interquartile range; HRmin: minimum heart rate; HRmax: maximum heart rate; HR1min: heart rate at 1 min post exercise; Lactate_max_: maximum plasma lactate concentration; Vmax: maximum speed reached by the treadmill; Delta-HR: difference between the maximum heart rate and the heart rate recorded at 1 min post-exercise.

**Table 4:** Demographic, cardiac and performance data of horses grouped according to the “severe arrhythmias” classification.

**Variable**		**“Severe Arrhythmias” Horses**		**No “Severe Arrhythmias” Horses**	
		**n**	**%**	**n**	**%**
Sex	female	11	16	57	84
	gelding	1	10	9	90
	male	11	14	69	86
**Variable**		**mean ± SD**	**median (IQR)**	**mean ± SD**	**median (IQR)**
Age (y.o)		-	3 (2)	-	3 (1)
HRmin (bpm)		30 ± 4	30 (7)	-	32 (6)
HRmax (bpm)		228 ± 8	228 (9)	-	232 (11)
HR1min (bpm)		153 ± 22	153 (32)	154 ± 18	154 (11)
Lactate_max_ (mmol/L)		26.84 ± 7.46	26.84 (9)	24.51 ± 6.41	24.51 (8)
Vmax (m/s)		-	11 (1)	-	11 (1)
Time to fatigue (s)		-	190 (70)	-	190 (72)
Delta-HR (bpm)		75 ± 22	75 (27)	76 ± 18	76 (20)

The table reports the number and percentage of horses with severe arrhythmias and horses with no severe arrhythmias (categorical variables), the mean and standard deviation values (continuous variables normally distributed), and the median and interquartile range (continuous variables normally and non-normally distributed). n.: number of horses; %: percentage of horses; SD: standard deviation; IQR: interquartile range; HRmin: minimum heart rate; HRmax: maximum heart rate; HR1min: heart rate at 1 min post exercise; Lactate_max_: maximum plasma lactate concentration; Vmax: maximum speed reached by the treadmill; Delta-HR: difference between the maximum heart rate and the heart rate recorded at 1 min post exercise.

**Table 5:** Results of the final multiple logistic regression model referred to the “at least one” and “severe arrhythmias” classification.

**“At Least One” Classification**			
**Variable**	***p* Value**	**OR**	**95% CI**
Age	0.08	1.24	0.98–1.60
**“Severe Arrhythmias” Classification**			
**Variable**	***p* Value**	**OR**	**95% CI**
HR_min_	**0.04**	0.89	0.78–0.98
Lactate_max_	0.08	1.07	0.99–1.15

OR: odd ratio; CI: confidence interval; HRmin: minimum heart rate; Lactate_max_: maximum plasma lactate concentration.

## Data Availability

The data presented in this study are available on request from the corresponding author.
